# The Effect of Ambient Air Pollution on Sperm Quality

**DOI:** 10.1289/ehp.0901022

**Published:** 2009-09-18

**Authors:** Craig Hansen, Thomas J. Luben, Jason D. Sacks, Andrew Olshan, Susan Jeffay, Lillian Strader, Sally D. Perreault

**Affiliations:** 1 National Center for Environmental Assessment, Office of Research and Development, U.S. Environmental Protection Agency, Research Triangle Park, North Carolina, USA; 2 Department of Epidemiology, University of North Carolina School of Public Health, Chapel Hill, North Carolina, USA; 3 Office of the Assistant Administrator, Office of Research and Development, U.S. Environmental Protection Agency, Research Triangle Park, North Carolina, USA

**Keywords:** air pollution, male reproductive system, O_3_, ozone, particulate matter, sperm quality

## Abstract

**Background:**

Research has suggested an association with ambient air pollution and sperm quality.

**Objectives:**

We investigated the effect of exposure to ozone (O_3_) and particulate matter < 2.5 μm in aerodynamic diameter (PM_2.5_) on sperm quality.

**Methods:**

We reexamined a previous cohort study of water disinfection by-products to evaluate sperm quality in 228 presumed fertile men with different air pollution profiles. Outcomes included sperm concentration, total sperm per ejaculate (count), and morphology, as well as DNA integrity and chromatin maturity. Exposures to O_3_ and PM_2.5_ were evaluated for the 90–day period before sampling. We used multivariable linear regression, which included different levels of adjustment (i.e., without and with season and temperature) to assess the relationship between exposure to air pollutants during key periods of sperm development and adverse sperm outcomes.

**Results:**

Sperm concentration and count were not associated with exposure to PM_2.5_, but there was evidence of an association (but not statistically significant) with O_3_ concentration and decreased sperm concentration and count. Additionally, a significant increase in the percentage of sperm cells with cytoplasmic drop [β = 2.64; 95% confidence interval (CI), 0.21–5.06] and abnormal head (β = 0.47; 95% CI, 0.03–0.92) was associated with PM_2.5_ concentration in the base model. However, these associations, along with all other sperm outcomes, were not significantly associated with either pollutant after controlling for season and temperature. Overall, although we found both protective and adverse effects, there was generally no consistent pattern of increased abnormal sperm quality with elevated exposure to O_3_ or PM_2.5._

**Conclusions:**

Exposures to O_3_ or PM_2.5_ at levels below the current National Ambient Air Quality Standards were not associated with statistically significant decrements in sperm outcomes in this cohort of fertile men. However, some results suggested effects on sperm concentration, count, and morphology.

Ambient air pollution has been associated with a variety of health effects, ranging from subclinical outcomes to death (Chen et al. 2008; Pope 2007). More recently, the effects of air pollution on reproductive and birth outcomes have garnered increased interest (Glinianaia et al. 2004; Šrám et al. 2005; Wang and Pinkerton 2007). However, a limited amount of research has been conducted to examine the association between air pollution and male reproductive outcomes, specifically semen quality, which includes sperm count and concentration along with morphologic and chromatin abnormalities.

A limited number of animal toxicologic studies have provided preliminary evidence of associations between exposure to air pollutants and semen quality outcomes. Associations have been observed between total air pollution and reduced daily sperm production in mice and rats receiving *in utero* or prenatal exposure to total diesel exhaust and filtered exhaust (Ono et al. 2007; Watanabe 2005). These observations are not limited to exposure durations timed to occur before or after birth, but also have been observed in adult mice exposed to diesel exhaust for up to 6 months (Yoshida et al. 1999).

To date, few epidemiologic studies have examined the association between air pollution and semen quality. These studies have considered various exposure durations before semen collection that encompass either the entire period of spermatogenesis (i.e., 90 days) or key periods of sperm development that correspond to epididymal storage, development of sperm motility, and spermatogenesis (i.e., 0–9, 10–14, and 70–90 days, respectively, before sample collection) (Johnson et al. 1997).

The initial epidemiologic studies that focused on male reproductive outcomes were conducted as part of the Teplice Program (Šrám et al. 1996), which examined many health outcomes, including semen quality (sperm numbers, motility, morphology, and chromatin), associated with episodically high ambient air pollution in Teplice, a heavily polluted area of the Czech Republic. Because the concentration of individual air pollutants covaried, these studies did not include single pollutant analyses but instead focused on a mix of air pollutants that included particulate matter ≤ 10 μm in aerodynamic diameter (PM_10_), PM total suspended particles (TSP), sulfur dioxide (SO_2_), nitrogen oxides (NO_x_), and carbon monoxide (CO). Source studies showed that air pollution resulted largely from the combustion of high-sulfur coal used for industry and home heating and that levels during winter episodes approached or exceeded air quality standards (Šrám 2001). Men 18 years of age residing in the heavily polluted district of Teplice were found to be at greater risk of having abnormalities in sperm morphology and chromatin integrity than men of similar age residing in Prachatice, a less polluted district (Selevan et al. 2000; Šrám et al. 1999). A follow-up longitudinal study conducted on a subset of the same men from the polluted district (Teplice) when they were 19–22 years of age revealed associations between total episodic air pollution (i.e., semen collection periods when air pollution levels were high vs. low) and abnormalities in sperm chromatin (Rubes et al. 2005). None of the Teplice Program studies (Rubes et al. 2005; Selevan et al. 2000; Šrám et al. 1999) found an association between air pollution and sperm production (total sperm count or concentration).

More recent studies conducted in the United States have also reported associations between ambient air pollution and sperm quality, but for individual pollutants [i.e., O_3_ and PM_2.5_ (particulate matter ≤ 2.5 μm in aerodynamic diameter)]. In a repeated-measures study in Los Angeles, California, Sokol et al. (2006) reported a reduction in average sperm concentration during three exposure windows (0–9, 10–14, and 70–90 days before semen collection) associated with high ambient levels of O_3_ in healthy sperm donors. In Salt Lake City, Utah, fine particulate matter (PM_2.5_) was associated with decreased sperm motility and morphology in clinical semen samples (Hammoud et al. 2009).

Taken together, the Teplice Program and U.S.-based studies suggest that exposure to ambient air pollution may result in a reduction in sperm quality; however, these studies are not directly comparable because of differences in the air pollution mix and sources, age and status of study populations, analytical methods employed across studies, and the pollutants and sperm parameters examined. We examined potential associations between ambient air pollution and sperm quality in fertile men using semen data from a recent cohort study (Luben et al. 2007; Olshan et al. 2007) that was designed to examine potential associations between exposure to disinfection by-products in tap water and male reproductive health. Only a few studies have examined the association of ambient air pollution with sperm parameters, and this is the first to do so in multiple geographic locations and among a group of fertile men. Semen outcomes (sperm number, morphology, and chromatin structure) were available for men residing in three counties in the southeastern United States. We obtained publically available air pollution data in these locations and examined the PM_2.5_ and O_3_ concentrations for potential associations with semen quality, considering four exposure windows that represent relevant stages of spermatogenesis (i.e., 0–9, 10–14, 70–90, and 0–90 days before semen collection).

## Materials and Methods

### Study design and subject recruitment

The design of the present study, The Healthy Men Study, has been described previously (Olshan et al. 2007). The University of North Carolina School of Public Health’s Institutional Review Board approved the study protocol, and all study participants gave written informed consent. Briefly, The Healthy Men Study identified male partners of pregnant women who participated in a prospective study of drinking water disinfection by-products and spontaneous abortion [the Right From the Start (RFTS) study] (Promislow et al. 2004; Savitz et al. 2005, 2006). Men were prospectively identified from the RFTS study and recruited from the three RFTS study sites (Wake County, NC; Shelby County, TN; Galveston County, TX). Men eligible for this study were 18–40 years of age. Each participant provided a reproductive history, but only men who had undergone a vasectomy or chemotherapy were excluded.

### Questionnaire

A computer-assisted telephone interview was administered to each participant by experienced interviewers, with responses entered directly into a computerized database (Luben et al. 2007). The average duration of the interview was approximately 40 min. Questions covered the following topics: general lifestyle, health, reproductive history, environment, diet, stress, occupational exposures, hobbies, and demographic factors.

### Semen collection and analyses

The methods for semen collection and analyses have been described in detail previously (Luben et al. 2007). Participants were asked to provide a single semen sample using a special kit designed to allow the man to collect a semen specimen in the privacy of his own home and at a time convenient to him (Royster et al. 2000). Before sending the kit to the participant, study staff confirmed by telephone the participant’s mailing address, gave brief instructions on how to use the kit, and asked the participant if he had previously used a similar collection method. Verbal instructions included the importance of doing the collection after 2–7 days of abstinence from sexual activity (Luben et al. 2007). A pilot study confirmed the stability of semen outcomes after simulated overnight shipping at 70°F or 40°F. Under all conditions, results were comparable except those for the sperm chromatin structure assay–DNA fragmentation index (SCSA-DFI), which increased significantly after shipping at 70°F but not 40°F (Perreault SD, unpublished data). Therefore, samples were shipped overnight with cold packs. The instructions accompanying the kit included photographs and instructions on how to properly collect the specimen; package the sample with cold packs and prepare it for shipping; and call to arrange the courier pickup (Luben et al. 2007). If the initial specimen volume was very low (< 0.5 mL), the man reported spillage or incomplete sample collection, shipping was delayed or the sample was not packaged correctly, or the participant’s abstinence interval was too far outside of the suggested 2- to 7-day range, participants were asked to provide a second or third specimen. This affected 10% (*n* = 20) of participants, and all 20 complied with the repeat collections.

All samples were processed upon receipt at a spermatology laboratory in the Reproductive Toxicology Division of the U.S. Environmental Protection Agency’s National Health and Environmental Effects Research Laboratory (Research Triangle Park, NC) by technicians trained in human semen analysis. All semen analysis protocols included quality control charts and competency review.

Immediately upon receipt, semen volume was measured and aliquots removed for determination of sperm concentration by IVOS-IDENT (Integrated Visual Optical System; Hamilton Thorne Research, Beverly, MA) (Zinaman et al. 1996) and calculation of total sperm count. Smears prepared from additional aliquots were air-dried and stored for later analyses of sperm morphology [World Health Organization (WHO) 1999]. Sperm motility, which declines over time and is therefore not a reliable measure for shipped semen, was not included in the statistical analysis. However, sperm motility and viability (using propidium iodide as a vital stain) were monitored. All samples retained motile and viable sperm, an indication that the sample had been collected and shipped according to instructions. Samples with low volume (< 0.5 mL) or evidence of spillage (or if the man reported incomplete collection) were discarded and another sample requested.

Additional aliquots (0.1 mL) were frozen and stored at −70°C for later analysis of chromatin integrity by the SCSA (Evenson and Jost 2000) and for chromatin maturity by chromomycin A3 (CMA) staining (Sakkas et al. 1995). For the SCSA, aliquots were shipped on liquid nitrogen to SCSA Diagnostics (Brookings, SD) for analysis according to established methods (Evenson et al. 2002). SCSA software calculates the percentage of sperm with fragmented DNA (%DFI). SCSA has been shown to be a highly reproducible test to measure %DFI, which compares favorably with other tests of sperm DNA damage such as the TUNEL (terminal deoxynucleotidyl transferase dUTP nick end labeling) and comet assays (Lewis and Agbaje 2008). The CMA assay is based on the stainability of sperm with CMA3, which detects sperm deficient in protamine as a characteristic of immaturity (Bianchi et al. 1993). We considered sperm to be CMA3 positive when at least 50% of the area of the nucleus fluoresced above background. Clinical studies have shown an association between relatively high percentages of CMA3 staining and subfertility or infertility (Bianchi et al. 1993; Esterhuizen et al. 2000). In both assays, aliquots of pooled semen were included in each run to serve as an internal standard.

We examined nine sperm outcomes reflective of testis function: *a*) sperm count (millions), *b*) sperm concentration (millions per milliliter), *c*) sperm morphology (percent normal sperm), *d*) percent of sperm cells with abnormal head, *e*) percent of sperm cells with abnormal midsection, *f* ) percent of sperm cells with abnormal tails, *g*) percent of sperm cells with cytoplasmic droplets, *h*) percent sperm with DNA fragmentation according to SCSA, and *i*) percent immature sperm according to CMA staining.

### Air pollution data and exposure assessment

We obtained air pollution data from the U.S. Environmental Protection Agency (EPA) Air Quality System Data Mart (U.S. EPA 2008) for the period of exposure in each of the three counties where the study subjects resided. Air pollution data were originally obtained for PM_10_, PM_2.5_, O_3_, nitrogen dioxide (NO_2_), SO_2_, and CO. However, in this study we focused on exposure to PM_2.5_ and O_3_ because the data for these pollutants were the most complete across all three counties for these pollutants (data for CO, NO_2_, and SO_2_ were monitored at only two of the three study sites during the study period). Meteorologic data were also obtained in the form of the daily minimum and maximum temperature readings.

For PM_2.5_, the data represent the 24–hr average, which was collected at two monitoring sites in Wake County, North Carolina (recorded daily at one site and every third day at the other), four monitors in Shelby County, Tennessee (two recorded daily and two every third day), and one monitor in Galveston County, Texas (recorded every third day). For O_3_ (reported as parts per billion), the data represent the maximum 8–hr average, which was collected at four monitors in Wake County and at two monitors each in Shelby County and Galveston County (all sites recorded daily data). For the daily maximum temperature, data were recorded at six monitors in Wake County, five monitors in Shelby County, and three monitors in Galveston County.

To allow for more complete exposure data, we interpolated the values for the 2 missing days between each 3-day reading for the monitors that measured PM_2.5_ concentration every third day. We used the PROC EXPAND procedure (version 9.1; SAS Institute Inc., Cary, NC) in which the successive nonmissing values were connected with straight lines (using the JOIN method). However, there were several periods in which a 3-day reading was missing, which created periods of > 2 days of missing data. The missing values within these periods were left as missing because we determined that it was too long a period to interpolate the data.

To estimate the daily level of PM_2.5_, O_3_, and maximum temperature within each county, where possible, we calculated an average across the multiple monitoring sites within the county; otherwise, the daily reading was obtained from one monitor within that county (e.g., PM_2.5_ in Galveston County). The estimated air pollution and temperature time series within each county was then linked to the 90–day period before semen sampling for each subject. For PM_2.5_ and O_3_, we then calculated an average exposure over the windows that represent important points of spermatogenesis (i.e., 0–9, 10–14, 70–90, and 0–90 days before sampling). We used the daily maximum temperature, rather than the average temperature, over each exposure period to facilitate comparison with the results presented by Sokol et al. (2006); the temperature variable in this analysis represents the number of days > 90°F within each exposure period.

### Data analysis

We performed statistical transformations on several of the outcome variables to better approximate the normality assumption of the linear model. Specifically, we applied a natural log transformation to the sperm count and concentration variables, and an arc sine-root transformation to the percentages of normal sperm cells; sperm cells with abnormal head, midsection, or tail; and sperm cells with cytoplasmic droplets.

For interpretability, each of the outcome variables was standardized (after statistical transformation, if applied) such that both the SD and the variance were equal to 1. Thus, each regression coefficient provides an estimate of the effect in terms of a change in SD of the response variable.

We characterized the distribution of demographic, exposure, and other characteristics for all participants and by individual study site. We first examined differences in demographic characteristics across the three sites using a chi-square test of independence for the categorical variables and analysis of variance (ANOVA) on age when examined as a continuous variable. In addition, we conducted bivariate analyses for all covariates and exposure variables with each of the outcome variables.

We used linear regression to assess the association between each exposure variable and outcome, adjusting for potential confounders. We made an *a priori* decision to adjust for the same variables used in the model specified by Luben et al. (2007). Additionally, we included season and temperature in subsequent models to compare the results with those of Sokol et al. (2006).

For the main analyses, we used three linear regression models. The base model (model 1) consisted of the air pollution exposure metric adjusted for age (indicator variables with > 35 years as the reference), days abstaining (indicator variables with > 8 days as the reference), education (indicator variables with “some college” as the reference), and smoking (indicator variables with nonsmoker as the reference). The categories for the covariates are shown in Table 1. Model 2 included the base model plus season of the semen sample (indicator variables with “winter” as the reference). Model 3 was model 2 plus temperature (the number of days > 90°F during the exposure window). We chose the > 90°F cutoff arbitrarily to facilitate comparison with the results of Sokol et al. (2006). Several studies have suggested that testicular function is influenced by season (Gyllenborg et al. 1999; Levine et al. 1988, 1990, 1992); this may account at least in part for the reduction in spring births in regions with warm climates, although it is unclear if this effect is related entirely to temperature or if there may be some other seasonal component, such as photoperiod, that leads to this phenomenon. The air pollution exposure metric was entered into the models as a continuous variable, and the β–coefficients are presented for a 15–ppb increase in O_3_ and a 10–μg/m^3^ increase in PM_2.5_.

To be included in the final analyses, which examined the exposure period 0–90 days before sampling, the subject had to have at least 45 days (50%) of exposure data available. For PM_2.5_, 80% (*n* = 183) of subjects had data for all 90 days, 14% (*n* = 32) had 70–89 days of data, and 6% (*n* = 13) had < 70 days (minimum number of days, 58). For O_3_, 75% (*n* = 171) of subjects had data for all 90 days, 4% (*n* = 8) had 70–89 days of data, 7% (*n* = 16) had 50–69 days of data, 5% (*n* = 11) had 30–49 days of data, and 10% (*n* = 22) had < 30 days of data (6 subjects had zero days of data). For the inclusion criteria of 45 days, all subjects had at least 45 days of PM_2.5_, and 87% (*n* = 199) had at least 45 days of O_3_.

Similarly, for analyses that examined periods < 90 days (i.e., 0–9, 10–14, and 70–90 days before sampling), a subject was required to have at least 50% of available data. For the 0–9 day period each subject was required to have at least 5 days of data; for the 10- to 14-day period, 3 days; and for the 70- to 90-day period, 10 days.

## Results

Table 1 shows the demographic characteristics of the study participants. In total, sperm data were available from 228 subjects across the three counties, with Galveston County having the least number of subjects (*n* = 45). Univariate analyses showed no statistically significant difference across the counties for race, smoking status, body mass index (BMI), caffeine intake, and vitamin use. Subjects from Wake County were significantly older and had a higher education and income, whereas fewer subjects from Shelby County drank alcohol in the 3 months before sampling. The number of samples collected within each season varied across the three counties, with most samples collected during the summer in Galveston County, during the spring in Shelby County, and during autumn in Wake County. No semen samples were collected from men in Wake County during the spring.

Table 2 shows the descriptive statistics for PM_2.5_ and O_3_ levels at each site within the three counties. The average PM_2.5_ concentration during the study periods was highest in Wake County, followed by Shelby and Galveston counties. In contrast, the average O_3_ concentration was slightly higher in Shelby County compared with the other counties.

The mean (median) sperm concentration for the entire group was 114.23 (90.50) million/mL (Table 3) and did not differ by study site (data not shown). The current WHO reference value for sperm concentration is ≥ 20 million/mL (WHO 1999). In this group of men, < 5% had sperm concentrations < 20 million/mL. The mean (median) sperm count for the entire group was 362 (265) million. The mean ± SD percentage of normal sperm for all samples was 14.14 ± 5.84% (Table 2) and did not differ by study site (data not shown). The most recent WHO guidelines (WHO 1999) did not specify a reference value for this measure. Nevertheless, the guidelines note that as sperm morphology falls below 15% normal (using strict criteria for scoring sperm as normal), the fertilization rate *in vitro* decreases. It is notable that the mean percentage of normal sperm in our study population of presumed fertile men was just below this cut point for reduced fertility. The mean ± SD percentages of abnormal sperm head, midsection, and tail were 78.59 ± 7.41%, 22.71 ± 8.89%, and 22.24 ± 14.25%, respectively, and did not differ by study site (data not shown). The mean ± SD for percentage of sperm with a cytoplasmic drop was 1.55 ± 1.52%.

An examination of sperm parameters for exposure windows < 90 days were not statistically significantly different than those reported for the 0- to 90-day exposure window. As a result, we focused our analysis on the association between exposure to PM_2.5_ and O_3_ and sperm parameters 0–90 days before semen collection. Table 4 shows the results of multivariable linear regression by sperm quality parameter for the 0- to 90-day exposure window. The β-coefficients show the change in SD for the sperm parameter in relation to a 15–ppb increase in O_3_ and a 10–μg/m^3^ increase in PM_2.5_. We found no significant association between sperm concentration and sperm count and either air pollutant across all three models examined. However, O_3_ did show an inverse association with sperm concentration and sperm count, and this persisted in all three models, yet the results failed to reach statistical significance. The only statistically significant adverse effects observed were for an increase in the percentage of sperm with an abnormal head (β = 0.47), and the percentage of cytoplasmic droplets (β = 2.64) in response to an increase in PM_2.5_. These results did not persist after controlling for season (model 2) and both season and temperature (model 3). Conversely, all other statistically significant results suggested protective associations between PM_2.5_ and O_3_ and morphologic sperm parameters.

**Figure 1:**
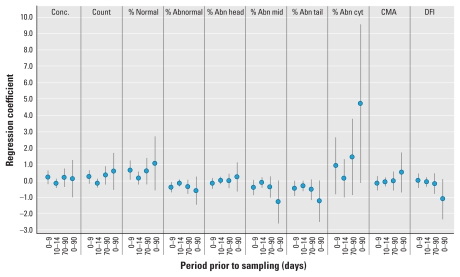
Results of multivariable linear regression by sperm quality parameter for the four exposure windows for PM_2.5_ (model 3). Abbreviations: Abn, abnormal; Conc., concentration; cyt, cytology; mid, midsection.

**Figure 2:**
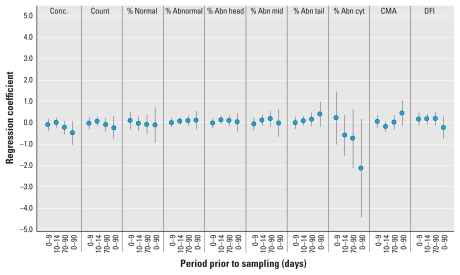
Results of multivariable linear regression by sperm quality parameter for the four exposure windows for O_3_ (model 3). Abbreviations: Abn, abnormal; Conc., concentration; cyt, cytology; mid, midsection.

Figures 1 and 2 summarize the results for all exposure windows for PM_2.5_ and O_3_, respectively, from model 3. We found no statistically significant associations between any sperm parameter and PM_2.5_ or O_3_.

## Discussion

In this study we examined potential associations between ambient air pollution and sperm quality among 228 men from three U.S. counties. Of the multiple analyses performed, the only statistically significant adverse association observed was between increased PM_2.5_ averaged over the 0– to 90–day period before semen sampling and an increase in the percentage of sperm with abnormally shaped heads and the percentage of sperm with cytoplasmic droplets. Neither of these results persisted after controlling for season and temperature.

Our results do not indicate a consistent pattern of association between O_3_ and PM_2.5_ and several measures of semen quality, but we did observe some similarities with previous studies. For example, in the first Teplice Program study, involving 408 men, Selevan et al. (2000) found that exposure to periods of elevated air pollution during the 90 days before semen sampling was associated with proportionately fewer motile sperm, fewer sperm with normal morphology, and, similar to our study, fewer sperm with a normal head shape. In the follow-up longitudinal study conducted on a subset of 37 of the same men from Teplice, episodes of high air pollution were associated with increased sperm DNA fragmentation, but not with abnormal morphology (Rubes et al. 2005). However pollution levels were lower in the second study. The authors hypothesized that carcinogenic components of PM may be responsible for the chromatin abnormalities, which are indicative of DNA damage in the sperm nuclei (Rubes et al. 2005). These studies did not include single pollutant analyses, but instead focused on a mix of air pollutants, which included PM_10_, PM-TSP, SO_2_, NO_x_, and CO. Neither of the two Teplice Program studies found an association between air pollution and sperm production (total sperm count or concentration).

The study by Sokol et al. (2006) provides an opportunity for comparisons with our study regarding associations between sperm concentration and sperm counts and O_3_ and PM_2.5_ as individual pollutants. Other sperm end points were not determined to be associated with O_3_ or PM_2.5_ in either study. The present study differed from that of Sokol et al. with respect to exposure assessment and semen collection [multiple semen samples were collected by Sokol et al. (2006), but we collected only one sample per participant]. In the Los Angeles study, Sokol et al. (2006) estimated personal exposure based on air pollution levels within the ZIP code of residence for each study participant, compared with the county average pollutant concentration from fixed-site monitors used in our study. They found a significant negative correlation between sperm concentration and O_3_ during the 0– to 9–, 10– to 14–, and 70– to 90–day periods before semen sampling. We also found evidence suggestive of an inverse association between O_3_ and sperm concentration and count, although the effect was not statistically significant, possibly because of our relatively smaller sample size. Also, our analyses used the maximum 8–hr average O_3_ concentration as the exposure metric, and we restricted the analysis to the O_3_ season, whereas Sokol et al. (2006) used a 24–hr average O_3_ exposure metric over the entire year. However, the O_3_ concentrations reported in the two studies are comparable.

Neither study found an association between PM_2.5_ and sperm concentration or count. Mean 24–hr average concentrations of PM_2.5_ ranged from 11 to 14 μg/m^3^ in the three counties examined in the present study, well below the 24–hr average regulatory standard of 35 μg/m^3^ set in 2006 (U.S. EPA 2009).

In a recent study conducted in Salt Lake City, Utah, Hammoud et al. (2009) evaluated 1,699 semen samples from 561 men and found a negative association between PM_2.5_, and sperm motility and sperm head morphology. This finding is similar to our result for sperm head morphology. The Utah study used a different methodology wherein both the sperm parameters and PM_2.5_ concentrations were averaged over the months and then the correlations for the 1–, 2–, and 3–month lag periods were analyzed. Individual exposures and characteristics were not assessed, and no other pollutants were investigated. Similar to the Teplice Program studies, Hammoud et al. (2009) found no association with sperm concentration in the Utah study.

In addition to these observational studies, several studies in occupational settings have supported an association between air pollution and decreased sperm quality. Decreased sperm motility, sperm count, and forward progression were found among motorway toll workers compared with a control group (De Rosa et al. 2003; Guven et al. 2008). Decreased sperm motility has also been found among traffic police, and this was associated with blood lead levels (Eibensteiner et al. 2005). In a study of coke-oven workers, Hsu et al. (2006) compared topside-oven workers with side-oven workers; based on personal monitoring and urinary samples, the topside-oven workers had significantly higher exposure to polycyclic aromatic hydrocarbons and higher rates of DNA-damaged sperm.

The biological mechanisms linking ambient air pollution to decreased sperm quality have yet to be determined. Sokol et al. (2006) identified several possible mechanisms, including O_3_-induced oxidative stress, inflammatory reactions, and the induction of the formation of circulating toxic species.

Despite the adverse associations in the present study between PM_2.5_ and sperm head morphology, and between O_3_ and sperm concentration and count, some results for PM_2.5_ and O_3_ indicate a protective effect on other sperm morphology parameters (e.g., abnormal midsection and tail). Because there is no biologically plausible explanation for protective effects on different regions of the sperm cell, these sperm morphology data should be interpreted with caution. Furthermore, the significant adverse association between PM_2.5_ and abnormal sperm head may have occurred by chance, given the large number of comparisons made for sperm morphology.

In addition to the small sample size of the present study, limitations include possible exposure misclassification as a consequence of estimating individual exposures from fixed-site monitors at the county level without knowledge of residential address or time–activity patterns of the study subjects. This would tend to underestimate any possible effect associated with air pollution (Rothman and Greenland 1998). The use of fertile men could also be considered a limitation because an adverse effect might be harder to detect in a group of healthy men. On the other hand, the use of men with questionable fertility (e.g., from infertility clinics) has its own disadvantages: In these men, poor semen quality may include abnormalities unrelated to air pollution exposure. Therefore, a random sample of men from the general population may encompass a more representative study population. An additional limitation of our study is the use of a single semen sample, which may not adequately represent a man’s testis function at any given time (Amann 2009) and does not allow for repeated measures on the same individual over times of varying exposures. Finally, we did not examine the effect of air pollution on an important indicator of sperm quality (i.e., sperm motility) due to the sperm collection methods used (WHO 1999).

## Conclusion

The present study provides suggestive evidence of an association between ambient air pollution and sperm quality. Results for PM_2.5_ suggest a possible adverse association with sperm head morphology and cytoplasmic droplets, although these results did not persist after controlling for season and temperature. Also, although not statistically significant, O_3_ was inversely associated with sperm concentration and count, consistent with results in Los Angeles (Sokol et al. 2006). Clearly, further research is needed, with better characterized exposure models, to explore this association in more detail.

## Figures and Tables

**Table 1:** Demographic characteristics of study participants by site [*n* (%)].

Covariate	All sites (*n* = 228)	Wake County, NC (*n* = 92)	Shelby County, TN (*n* = 91)	Galveston County, TX (*n* = 45)	*p*-Value (χ^2^)
Age (years)					0.023*

19–24	26 (11)	3 (3)	14 (15)	9 (20)	
25–29	69 (30)	24 (26)	31 (34)	14 (31)	
30–34	95 (42)	47 (51)	33 (36)	15 (33)	
35–40	38 (17)	18 (20)	13 (14)	7 (16)	

Race					0.660

Black	18 (8)	6 (7)	9 (10)	3 (7)	
Nonblack	210 (92)	86 (93)	82 (90)	42 (93)	

Ethnicity					< 0.001

Hispanic	9 (4)	2 (2)	0 (0)	7 (16)	
Non-Hispanic	219 (96)	90 (98)	91 (100)	38 (84)	

Education					< 0.001

High school only	35 (15)	3 (3)	18 (20)	14 (31)	
Some college	46 (20)	16 (17)	16 (18)	14 (31)	
Graduated college	147 (65)	73 (79)	57 (63)	17 (38)	

Income (US$/year)					0.002

≤ 40,000	52 (23)	10 (12)	25 (29)	17 (40)	
40,001–80,000	109 (48)	48 (52)	41 (45)	20 (44)	
≥ 80,001	64 (28)	33 (36)	24 (26)	7 (16)	

BMI					0.060

< 18.5 (underweight)	0	0	0	0	
18.5 to < 25 (normal)	63 (28)	30 (33)	23 (25)	10 (22)	
25 to < 30 (overweight)	108 (47)	47 (51)	41 (45)	20 (44)	
30 to < 35 (obese I)	34 (15)	11 (12)	12 (13)	11 (24)	
≥ 35 (obese II)	23 (10)	4 (4)	15 (16)	4 (9)	

Smoking status					0.116

Yes	93 (41)	32 (35)	37 (41)	24 (53)	
No	135 (59)	60 (65)	54 (59)	21 (47)	

Alcohol use					0.007

Yes	175 (77)	77 (84)	60 (66)	38 (84)	
No	53 (23)	15 (16)	31 (34)	7 (16)	

Days abstaining					0.016

2–3	84 (37)	38 (41)	36 (40)	10 (22)	
4–8	124 (54)	50 (54)	48 (53)	26 (58)	
> 8	20 (9)	4 (4)	7 (8)	9 (20)	

Caffeine intake (mg/day)					0.945

None	0	0	0	0	
> 0–150	169 (93)	64 (70)	64 (70)	41 (93)	
> 150–300	7 (4)	3 (3)	3 (3)	1 (2)	
> 300	6 (3)	3 (3)	2 (2)	1 (2)	
Missing	45 (20)	22 (24)	22 (24)	1 (2)	

Vitamin use					0.146

Yes	98 (43)	45 (49)	32 (35)	21 (47)	
No	130 (57)	47 (51)	59 (65)	24 (53)	

Season					< 0.001

Spring	43 (19)	0 (0)	31 (34)	12 (27)	
Summer	60 (26)	22 (24)	17 (19)	21 (47)	
Autumn	76 (33)	47 (51)	22 (24)	7 (16)	
Winter	49 (21)	23 (25)	21 (23)	5 (11)	

* *p* = 0.001 for differences across the three sites when examined as a continuous variable by ANOVA.

**Table 2:** Air pollution data for the three study sites.

	Wake County, NC, 25 April 2002 to 16 January 2003 (267 days)		Shelby County, TN, 9 January 2003 to 5 May 2004 (483 days)		Galveston County, TX, 9 January 2003 to 5 May 2004 (483 days)	
Pollutant	Mean ± SD	Range	Mean ± SD	Range	Mean ± SD	Range
PM_2.5_ (μg/m^3^)						
Site 1	14.1 ± 7.7	2.3–62.7	13.2 ± 5.1^a^	2.7–35.2	10.9 ± 4.0^a^	3.4–25.7
Site 2	14.2 ± 6.7^a^	3.5–46.1	12.0 ± 5.9	2.1–35.8		
Site 3			13.2 ± 6.2	3.1–38.0		
Site 4			11.3 ± 5.5^a^	2.6–34.1		
Average	14.2 ± 6.9	2.3–54.4	12.6 ± 5.1	3.5–35.2		
O_3_ (ppb)						
Site 1	31.5 ± 16.1	5.7–71.0	28.0 ± 9.3	2.0–55.1	34.8 ± 14.2	9.0–83.2
Site 2	35.5 ± 16.7	4.0–74.2	36.4 ± 10.3	2.0–70.6	25.1 ± 12.4	5.8–61.7
Site 3	36.8 ± 15.3	4.5–69.9				
Site 4	36.8 ± 16.3	3.9–75.1				
Average	30.8 ± 16.3	4.8–70.0	32.2 ± 9.5	2.0–57.7	30.5 ± 13.3	8.5–70.7

a Recorded every 3 days (otherwise daily).

**Table 3:** Distribution of outcome variables for all Healthy Men Study sites.

Outcome	No.	Mean ± SD	Median	Range
Sperm concentration (millions/mL)	225	114.2 ± 90.1	90.5	2.4–709.7
Sperm count (millions/sample)	225	362 ± 311	265	5–1,845
Percent normal morphology	228	14.1 ± 5.8	13.3	2.0–36.0
Percent abnormal morphology	228	85.9 ± 5.8	86.8	64.0–98.0
Percent abnormal head	228	78.6 ± 7.4	79.3	56.0–97.0
Percent abnormal midsection	228	22.7 ± 8.9	21.0	7.0–53.0
Percent abnormal tail	228	22.2 ± 14.3	18.3	2.0–65.0
Percent cytoplasmic droplets	228	1.6 ± 1.5	1.0	0.0–9.0
Percent CMA	223	60 ± 20	60	20–90
Percent DFI	190	20 ± 10	20	0–70

**Table 4:** Results of multivariable^a^ linear regression by sperm quality parameter for the 0- to 90-day exposure window.

	PM_2.5_ [β (95% CI)]			O_3_ [β (95% CI)]		
Parameter^b^	Model 1	Model 2	Model 3	Model 1	Model 2	Model 3
Sperm concentration^c^	−0.10 (−0.66 to 0.47)	0.16 (−0.53 to 0.86)	0.16 (−0.96 to 1.29)	−0.19 (−0.44 to 0.06)	−0.16 (−0.51 to 0.19)	−0.52 (−1.07 to 0.04)
Sperm count^c^	0.07 (−0.50 to 0.64)	0.36 (−0.34 to 1.05)	0.62 (−0.49 to 1.73)	−0.18 (−0.43 to 0.07)	−0.04 (−0.39 to 0.30)	−0.26 (−0.81 to 0.28)
Percent normal morphology^d^	0.96 (0.13 to 1.78)*	1.08 (0.06 to 2.10)*	1.09 (−0.55 to 2.73)	0.02 (−0.36 to 0.50)	0.26 (−0.27 to 0.79)	−0.15 (−0.98 to 0.68)
Percent abnormal morphology^d^	−0.50 (−0.93 to −0.07)*	−0.57 (−1.10 to −0.03)*	−0.57 (−1.43 to 0.29)	−0.01 (−0.21 to 0.19)	−0.14 (−0.41 to 0.14)	0.08 (−0.36 to 0.51)
Percent abnormal head^d^	0.47 (0.03 to 0.92)*	0.46 (−0.09 to 1.01)	0.27 (−0.62 to 1.16)	0.07 (−0.13 to 0.26)	0.18 (−0.09 to 0.44)	0.00 (−0.43 to 0.42)
Percent abnormal midsection^d^	−1.44 (−2.11 to −0.78)*	−1.55 (−2.36 to −0.74)*	−1.25 (−2.55 to 0.05)	−0.11 (−0.41 to 0.19)	−0.52 (−0.93 to −0.11)*	−0.05 (−0.68 to 0.59)
Percent abnormal tail^d^	−1.77 (−2.41 to −1.12)*	−1.80 (−2.58 to −1.02)*	−1.20 (−2.45 to 0.05)	−0.13 (−0.41 to 0.16)	−0.51 (−0.89 to −0.13)*	0.37 (−0.21 to 0.94)
Percent cytoplasmic droplets^d^	2.64 (0.21 to 5.06)*	2.93 (−0.07 to 5.92)	4.74 (−0.07 to 9.56)	−0.09 (−1.15 to 0.98)	−0.30 (−1.78 to 1.17)	−2.17 (−4.47 to 0.14)
Percent CMA	0.09 (−0.52 to 0.70)	−0.02 (−0.78 to 0.74)	0.55 (−0.66 to 1.76)	−0.02 (−0.29 to 0.25)	0.07 (−0.31 to 0.44)	0.41 (−0.17 to 0.99)
Percent DFI	−0.77 (−1.55 to 0.00)	−0.64 (−1.63 to 0.35)	−1.07 (−2.30 to 0.15)	−0.13 (−0.43 to 0.17)	−0.17 (−0.58 to 0.24)	−0.27 (−0.78 to 0.25)

CI, confidence interval. Model 1 is the base model; model 2 is the base model + season; and model 3 is the base model + season + temperature. The β-coefficients show the change in SD for the sperm parameter in relation to a 15-ppb increase in O_3_ and a 10-μg/m^3^ increase in PM_2.5_.

a Base model adjusted for age, days abstaining, education level, and smoking.

b All outcomes standardized such that SD = variance = 1.00.

c Natural log transformation applied.

d Arc sine-root transformation applied.

* *p* < 0.05.
